# Batroxobin in combination with anticoagulation may promote venous sinus recanalization in cerebral venous thrombosis: A real‐world experience

**DOI:** 10.1111/cns.13093

**Published:** 2019-01-23

**Authors:** Jia‐Yue Ding, Li‐Qun Pan, Yan‐Yu Hu, Gary B. Rajah, Da Zhou, Chao‐Bo Bai, Jing‐Yuan Ya, Zhong‐Ao Wang, Ke‐Xin Jin, Jing‐Wei Guan, Yu‐Chuan Ding, Xun‐Ming Ji, Ran Meng

**Affiliations:** ^1^ Departments of Neurology and Neurosurgery Xuanwu Hospital, Capital Medical University Beijing China; ^2^ Center of Stroke Beijing Institute for Brain Disorders Beijing China; ^3^ Center of Sleep Xianyue Hospital Xiamen China; ^4^ Department of Neurosurgery Wayne State University School of Medicine Detroit Michigan

**Keywords:** anticoagulation, batroxobin, cerebral venous thrombosis, defibrinogenating effect

## Abstract

**Aims:**

The objective of this study was to evaluate cerebral venous recanalization with magnetic resonance black‐blood thrombus imaging (MRBTI) in patients with cerebral venous thrombosis (CVT) who underwent batroxobin treatment in combination with anticoagulation.

**Methods:**

A total of 31 CVT patients were enrolled in this real‐world registry study. The patients were divided into batroxobin (n = 21) and control groups (n = 10). In addition to the same standard anticoagulation as in the control group, patients in the batroxobin group underwent intravenous batroxobin for a total of three times.

**Results:**

In the batroxobin group compared with the control group, we found better odds of recanalization degree [adjusted OR (95%CI) of 8.10 (1.61‐40.7)] and segment‐stenosis attenuation [adjusted OR (95%CI) of 4.48 (1.69‐11.9)] with batroxobin treatment. We further noted a higher ratio of patients with the attenuation of stenosis [adjusted OR (95%CI) of 26.4 (1.10‐635)]; as well as a higher ratio of segments with stenosis reversion [adjusted OR (95%CI) of 4.52 (1.48‐13.8)]. However, neurological deficits between the two groups showed no statistical difference at 90‐day follow‐up (*P* > 0.05).

**Conclusions:**

Batroxobin may promote venous sinus recanalization and attenuate CVT‐induced stenosis. Further randomized study of this promising drug may be warranted to better delineate the amount of benefit.

## 1 INTRODUCTION

Cerebral venous thrombosis (CVT) is an uncommon subtype of stroke with a highly variable clinical course including life‐threatening deterioration especially with multisinus involvement.^1^, ^2^, ^3^ Although anticoagulation (heparin and warfarin) is still recommended as the gold standard for CVT treatment per guidelines issued by the American Heart Association (AHA) and American Stroke Society (ASA) in 2011, and the European Federation of Neurological Societies (EFNS) in 2017,^1^, ^2^ the outcomes of CVT still can include deterioration despite aggressive anticoagulation^4^, ^5^; meanwhile, the safety and efficacy of some other interventions (such as systematic and endovascular thrombolysis or mechanical venous thrombectomy) of CVT are invasive and typically used when patients fail anticoagulation.^6^, ^7^, ^8^, ^9^, ^10^ Adjunctive noninvasive therapies are needed to increase the efficacy of anticoagulation for CVT.

Batroxobin is a thrombin‐like serine protease extracted from Bothrops atrox moojeni venom. It is a defibrinogenating agent which is more commonly used on cerebral arterial thrombosis.^11^, ^12^, ^13^, ^14^, ^15^ A recent study revealed that batroxobin in combination with anticoagulation may be safe and effective for reducing fibrinogen in cerebral venous circulation and promoting thrombolysis.^16^ The authors of the previous study utilized time‐of‐flight magnetic resonance venography (TOF MRV) in patients who underwent intravenous batroxobin plus anticoagulation,^16^ the study identified batroxobin as a promising agent in CVT control. Despite these positive findings, TOF MRV assesses recanalization only by indirect imaging of venous flow perturbation caused by the thrombus; thus, this method may be less specific as it cannot depict the dissolving thrombus and luminal patency within the cerebral vein and venous sinus.^2^, ^17^ Direct visualization of the thrombus itself is a problem overcome by magnetic resonance black‐blood thrombus imaging (MRBTI) technique. MRBTI is a 3‐dimensional variable flip angle turbo spin echo and is capable of achieving accurate detection of thrombus and patency status in cerebral venous system 18. Herein, with the help of MRBTI technique, we aimed to further evaluate the efficacy of batroxobin on promoting CVT recanalization.

## 2 METHODS

### 2.1 Patients collection

This is a real‐world patient registry study performed in accordance with the guidelines of the 1964 Declaration of Helsinki. Given the real‐world nature of our study, randomization was not possible. All procedures conducted in this study involving human participants have been approved by the Institutional Ethics Committee (Xuanwu Hospital, Capital Medical University). The informed consents were obtained from the patients before initiating any study‐specific procedures. From March 2011 through April 2018, a total of 31 patients who were confirmed as CVT in the neurology department of Xuanwu Hospital, Capital Medical University, were recruited into this study. The diagnosis of CVT was confirmed by MRBTI. The detailed criteria of diagnosis could be seen in the current CVT guidelines.^1^, ^2^ The enrolled patients were screened with the following inclusion and exclusion criteria:

Inclusion criteria: (a) The diagnosis of CVT should be confirmed; (b) Age ranged from 18 to 80; (c) anticoagulation was the main therapeutic strategy in hospital; and (d) MRBTI was finished prior to the treatment and performed again at long‐term follow‐up.

Exclusion criteria: (a) Patients underwent endovascular treatment or thrombolysis (such as recombinant tissue plasminogen activator and urokinase) prior to the admission or during hospitalization; (b) Subjects without complete data; (c) The levels of baseline plasma fibrinogen were equal to or less than 1 g/L at admission; and (d) Patients were afflicted by other severe diseases.

### 2.2 Intervention and assessment

According to batroxobin use or not, the cohort of patients was grouped into batroxobin (batroxobin in combination with anticoagulation) and control (anticoagulation alone). All of the eligible patients in the two groups underwent standard anticoagulation according to current CVT management guidelines [low molecular weight heparin (LMWH) subcutaneous injection (0.4 mg/q12 h) bridged with warfarin 3 mg/d for 5‐7 days, followed by oral warfarin 3 mg/d, to maintain the international normalized ratio (INR) fluctuating between 2 and 3]. In the batroxobin group, all patients underwent intravenous batroxobin after signed an informed consent [batroxobin injection (produced by Beijing Tuobixi Pharmaceutical Co., Ltd., the approval number was H20031074) 10 BU for the first time, followed by 5 BU every other day, for two times, unless the level of fibrinogen dropped to 1.0 g/L or less]. The paradigm aforementioned was also depicted in our previously published study.^16^

MRBTI was used to evaluate the stenosis extent in cerebral venous system in CVT patients. All magnetic resonance tests were conducted on a 3.0‐T system (MAGNETOM Verio, Siemens Healthcare, Erlangen, Germany) with a 32‐channel head coil for signal reception. Typical imaging parameters included oblique coronal single‐slab coverage, repetition time = 800 ms, echo time = 22 ms, matrix = 198 × 192, field of view = 160 × 200 mm^2^, slice thickness = 0.6‐1.0 mm, slices = 100‐200, and scan time = 6‐8 minutes. The venous stenosis extent presented on MRBTI corresponded to the grading criteria in cerebral arteries as normal (<30%), mild (30%‐49%), moderate (50%‐69%), severe stenosis (70%‐90%), and occlusion (100%).^19^, ^20^ In case of multistenosis in one segment, the most severe section was evaluated. Imaging assessment was finished by two independent readers with extensive experience.

### 2.3 Outcome evaluation

**Figure 1:**
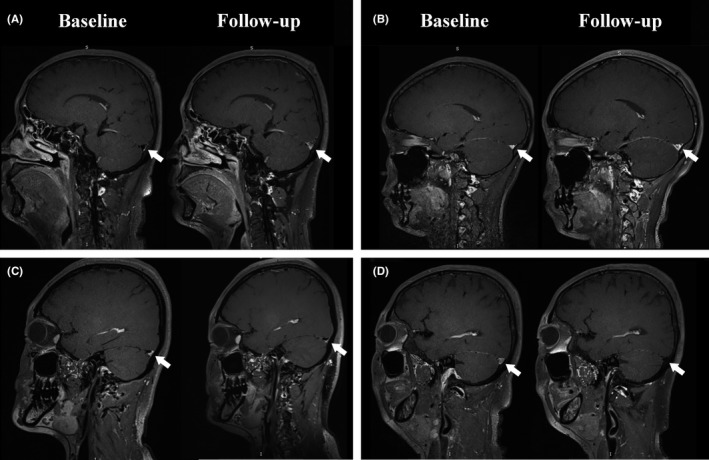
On MRBTI maps (follow‐up vs. baseline), the degree of the segment‐stenosis attenuation consists of: A, Class 0: exacerbation (thrombus increased); B, Class 1: no change or mild recovery (thrombus no change or reduced <30%); C, Class 2: moderate recovery (thrombus reduced to 30%‐60%); D, Class 3: obvious or complete recovery (thrombus reduced to 60%‐100%)

The recanalization degree on TOF MRV and the degree of the segment‐stenosis attenuation on MRBTI were followed up and recorded as the primary outcome. The recanalization degree was graded into four classes,^21^ Class 0: complete nonrecanalization; Class 1: partial recanalization of one or more occluded sinuses with improved collateral flow; Class 2: complete recanalization of one sinus but persistent occlusion of the other sinuses; and Class 3: complete recanalization of all occluded sinuses. Of the four classes, Class 2 and 3 were identified as recanalization, while Class 0 and 1 were defined as nonrecanalization. The stenosis attenuation was ranked into four classes according to the variation of stenosis extent from baseline to follow‐up on MRBTI maps (Figure 1), Class 0: exacerbation (thrombus increased); Class 1: no change or mild recovery (thrombus reduced <30%); Class 2: moderate recovery (thrombus reduced to 30%‐60%); and Class 3: obvious or complete recovery (thrombus reduced to 60%‐100%).

The secondary outcomes comprised the ratio of patients with recanalization measured by TOF MRV, and the ratio of patients with stenosis attenuation, as well as the ratio of segments with stenosis amelioration, and the stenosis extent per involved segments. All of which were assessed by MRBTI, furthermore, the neurological deficits including the National Institute of Health Stroke Scale (NIHSS) scores at discharge, the modified Rankin Scale (mRS) scores at 3 months, and hemorrhage occurrence/aggravation during hospitalization were also recorded. The stenosis attenuation was defined as stenosis with a ≥30% improvement in vessel diameter. The hemorrhage aggravation was an increase of 3 mL or >33% of primary hematoma volume on the follow‐up images.^22^

### 2.4 Statistical analysis

SPSS 19.0 was used for data analysis. Continuous data following a Gaussian distribution were presented as mean ± standard deviation and analyzed with t test; otherwise, it was expressed as median (interquartile range, IQR) and analyzed with a Mann‐Whitney *U* test. Categorical data were processed by chi‐square test (for dichotomous variable) or Mann‐Whitney *U* test (for ordinal dependent variable). For further test, multivariate analyses such as logistic regression model and linear regression models were needed to rule out confounding effects. The results were displayed as an odd ratio (OR) along with 95% confidence interval (95%CI). *P* < 0.05 was considered indicative of statistical significance.

## 3 RESULTS

### 3.1 Baseline characteristics

A total of 31 subjects were recruited in this study, 21 patients in the batroxobin group and 10 cases in the control group. Thrombus was present in a total of 92 segments (35 transverse sinus, 22 sigmoid sinus, 21 sagittal sinus, 2 deep veins, 1 straight sinus, and 11 cortical veins), among which, 64 segments were in the batroxobin group and 28 in the control group. Overall, the average age was 32.8 ± 16.7 years, the mean time from symptom onset to admission was 21.5 ± 16.9 days, the mean hospital stay was 12.7 ± 2.83 days, and the mean imaging follow‐up interval was 153 ± 114 days. All subjects were alive during either hospitalization or long‐term follow‐up, including one patient in the control group who had a cerebral hemorrhage at admission. The baseline data (including demographic, risk factors, imaging assessment, and neurological deficits) were equivalent between the two cohorts, except for hyper‐fibrinogen (*P* = 0.03). Details are displayed in Table 1.

**Table 1:** Characteristics of involved subjects at baseline

	Batroxobin, n = 21	Control, n = 10	*P*‐value
Demographic			
Gender (Female/Male)	12/9	4/6	0.458
Age (y)	29.8 ± 14.5	39.2 ± 21.5	0.226
Clinical appearances			
Coma	2 (9.5%)	0 (0.0%)	1.000
Seizures	12 (57.1%)	3 (30.0%)	0.252
Focal signs	9 (42.9%)	2 (20.0%)	0.262
Hyper‐fibrinogen condition	9 (42.9%)	0 (0.0%)	0.030
NIHSS scores at admission	0.00 (0.00, 2.00)	0.00 (0.00, 2.50)	0.539
mRS scores at admission	3.00 (2.00, 3.00)	2.00 (1.00, 4.00)	0.370
Time of hospital stay (d)	13.14 ± 2.89	11.90 ± 2.64	0.260
Time from symptom onset to admission (d)	19.84 ± 15.28	24.60 ± 20.04	0.480
Follow‐up time	132.67 ± 113.23	194.50 ± 110.58	0.163
Intracerebral hemorrhage	0 (0.0%)	1 (10.0%)	0.323
Risk factors			
Pregnancy	3 (14.3%)	1 (10.0%)	1.000
Infection	3 (14.3%)	1 (10.0%)	1.000
Oral contraceptives	2 (9.5%)	0 (0.0%)	1.000
Hyperhomocysteinemia	4 (19.0%)	0 (0.0%)	0.277
Thrombophilia	9 (42.9%)	3 (30.0%)	0.697
Hematologic disorders	2 (9.5%)	3 (30.0%)	0.296
Systemic diseases	2 (9.5%)	0 (0.0%)	1.000

### 3.2 Primary outcomes

According to the recanalization criteria previously mentioned using TOF MRV, the recanalization degree at follow‐up in batroxobin group was profoundly higher than that in control, regardless of adjusting for confounders (follow‐up time and thrombophilia) or not [unadjusted OR (95%CI) of 8.22 (1.73‐39.1), *P* < 0.01; adjusted OR (95%CI) of 8.10 (1.61‐40.7), *P* = 0.01]. Further subgroup analysis of patients with normal fibrinogen (1 g/L < fibrinogen<4 g/L) revealed the batroxobin group again had better recanalization degree [OR (95% CI) 5.64 (1.06‐30.0) and adjusted OR (95%CI) was of 5.65 (0.95‐33.4)]. Hyper‐fibrinogen levels were included only in batroxobin group in this study.

**Figure 2:**
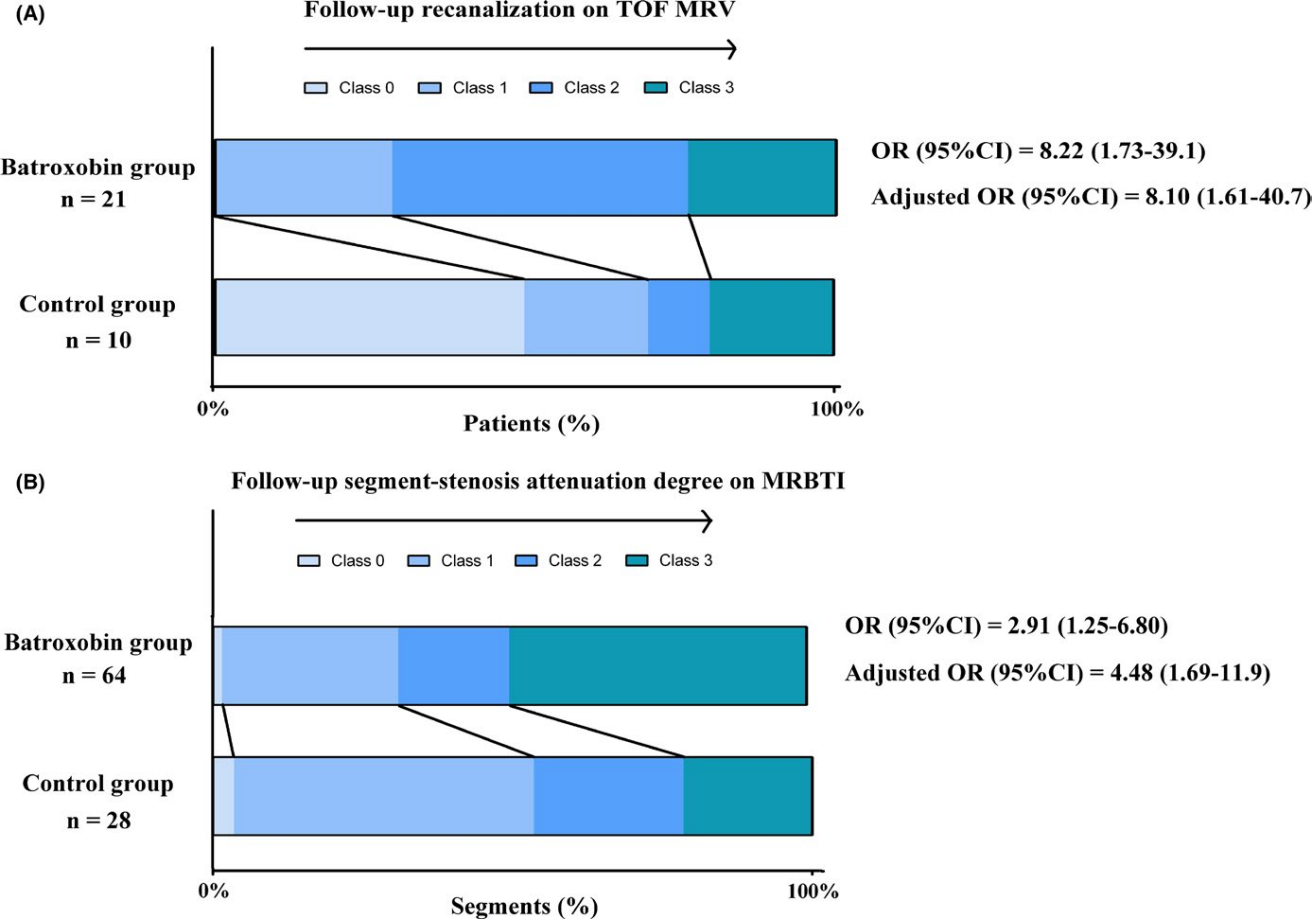
Batroxobin group vs. control: The raw distribution of (A) the follow‐up recanalization; (B) the segment‐stenosis extent attenuation

When evaluated by MRBTI follow‐up, segment stenosis reversed more profoundly in batroxobin group compared with control [unadjusted OR (95%CI) of 2.91 (1.25‐6.80), *P* = 0.01; adjusted OR (95%CI) of 4.48 (1.69‐11.9), *P* < 0.01]. Among the patients with normal fibrinogen, the segment‐stenosis attenuation in batroxobin group was more obvious than that in control [OR (95%CI) of 3.35 (1.33‐8.43), *P* = 0.01; adjusted OR (95%CI) of 5.08 (1.67‐15.5), *P* < 0.01]. All of above are displayed in Figure 2.

### 3.3 Imaging outcomes

Of the 31 subjects enrolled, 15/21 cases (71.4%) in the batroxobin group displayed recanalization on TOF MRV, while only 3/10 (30.0%) of the control patients had similar findings [unadjusted OR (95%CI) of 5.84 (1.12‐30.4); adjusted OR (95%CI) of 6.05 (1.06‐34.7)]. Moreover, there were 90.5% (19/21) cases in batroxobin group presenting with stenosis improving ≥30% on the follow‐up MRBTI maps, while only 60.0% (6/10) in control showed stenosis improvement on imaging [unadjusted OR (95%CI) of 6.33 (0.92‐43.6); adjusted OR (95%CI) of 26.4 (1.10‐635)]. Similarly, of the 92 involved stenotic segments, the ratio of segments with stenosis extent improvement (≥30%) was 68.8% (44/64) in batroxobin group, but 46.4% (13/28) in control [unadjusted OR (95%CI) of 2.54 (1.02‐6.32); adjusted OR (95%CI) of 4.52 (1.48‐13.8)]. In contrast, there was no difference on stenosis extent in the diseased segments at baseline between the two groups [batroxobin versus control: unadjusted OR (95%CI) of 0.94 (0.43‐2.08); adjusted OR (95%CI) of 0.66 (0.28‐1.57)]. The results are presented in Table 2.

**Table 2:** Secondary outcomes in the 31 patients

Secondary outcomes	Batroxobin group (Patient/segment, n = 21/64)	Control group (Patient/segment, n = 10/28)	Unadjusted OR (95%CI)	Adjusted OR (95%CI)
Follow‐up evaluation on TOF MRV				
Patients with recanalization, n (%)	15 (71.4)	3 (30.0)	5.84 (1.12‐30.40)^a^	6.05 (1.06‐34.69)^a^
Follow‐up evaluation on MRBTI				
Patients with stenosis relieved, n (%)	19 (90.5)	6 (60.0)	6.33 (0.92‐43.62)	26.36 (1.10‐634.65)^a^
Segments with stenosis relieved, n (%)	44 (68.8)	13 (46.4)	2.54 (1.02‐6.32)^a^	4.52 (1.48‐13.75)^b^
Stenosis extent, median (IQR)	2.00 (1.00, 3.00)	2.00 (1.00, 3.00)	0.94 (0.43‐2.08)	0.66 (0.28‐1.57)
Neurological deficits				
NIHSS at discharge, median (IQR)	0.00 (0.00, 1.00)	0.00 (0.00, 2.00)	0.71 (0.12‐4.26)	0.61 (0.34‐1.10)
mRS at 3 mo, median (IQR)	2.00 (1.00, 2.00)	2.00 (1.00, 2.25)	1.10 (0.27‐4.49)	1.14 (0.26‐5.04)
Hemorrhage occurrence/aggravation, n (%)	0 (0.0)	0 (0.0)	—	—

—: nonavailable.

Binary data or ordinal data were analyzed through a binary or ranked logistic regression model to adjust for confounding effects, while continuous data analysis was performed with a linear regression model to adjust for confounding effects. The confounding factors included follow‐up time and thrombophilia in the multivariate analyses of patients with stenosis relieved, segments with stenosis relieved and stenosis extent, and NIHSS at admission in the analysis of NIHSS at discharge and mRS at 6 mo.

a *P* < 0.05.

b *P* < 0.01.

### 3.4 Neurological deficits

No difference was detected between the two groups regarding both NIHSS scores at discharge and mRS scores at 3 months for the batroxobin group versus control group: OR (95%CI) for NIHSS score improvement was of 0.71 (0.12‐4.26) and 0.61 (0.34‐1.10) after adjustment and OR (95%CI) for mRS scores was of 1.10 (0.27‐4.49) and 1.14 (0.26‐5.04) after adjustment. There were no cerebral hemorrhage events (emerging hemorrhage or aggravating hemorrhage) during patient hospitalization. The results aforementioned are shown in Table 3.

**Table 3:** The follow‐up changes of thrombus on each subtype of segments in batroxobin group vs control

	Afflicted patients (num.)	Stenosis extent	Segments with stenosis relieved	Stenosis attenuation degree
Superior sagittal sinus	20	0.99 (0.12‐8.43)	11.68 (0.66‐206.72)	17.98 (1.26‐256.91)^a^
Inferior saggital sinus	1	—	—	—
Left transverse sinus	15	0.50 (0.06‐4.54)	1.24 (0.09‐16.30)	2.86 (0.28‐29.16)
Right transverse sinus	20	0.52 (0.07‐3.77)	12.49 (0.39‐395.63)	7.28 (0.38‐140.97)
Left sigmoid sinus	10	9.84 (0.47‐204.00)	5.37 (0.04‐709.33)	2.72 (0.21‐35.75)
Right sigmoid sinus	12	0.60 (0.03‐10.65)	4.40 (0.13‐145.99)	2.33 (0.07‐79.68)
Deep veins	2	—	—	—
Straight sinus	1	—	—	—
Cortical veins	11	0.10 (0.00‐6.38)	0.32 (0.01‐12.94)	4.38 (0.08‐227.33)

—: nonavailable.

Ranked logistic regression model for stenosis extent and stenosis attenuation degree, and binary logistic regression model for number of segments with stenosis relieved are used to adjust for confound effect (follow‐up time and thrombophilia). The results are expressed as adjusted OR (95% CI).

a *P* < 0.05.

### 3.5 Thrombus outcomes in different venous sinuses

The stenosis extent and the ratio of segments with stenosis attenuation in different venous sinuses involved at baseline were not statistically different between the two groups. The degree of segment‐stenosis attenuation in superior sagittal sinus in batroxobin group was more remarkable than that in control [adjusted OR (95%CI) of 18.0 (1.26‐257), *P* = 0.03]; however, these results were not seen in other venous sinus involvement.

## 4 DISCUSSION

Coagulation abnormalities are present by a number of conditions, including surgery, pregnancy, puerperium, trauma, anticardiolipin syndrome, malignancy, and thrombophilia. These conditions are considered to be involved mechanisms contributing to CVT.^1^, ^2^ To overcome the imbalance in hypercoagulability in CVT, the standard therapy is anticoagulation.^1^, ^2^ At times anticoagulation alone is not sufficient to reverse the disease process of CVT despite favorable clinical efficacy.^2^, ^23^, ^24^ We sought to identify adjunctive noninvasive therapies for multitarget treatments to coagulation‐fibrinolysis pathways. Our hope was to identify a synergistic agent that can be utilized with anticoagulation for CVT recanalization.

As we all know, only with the help of AT‐III, can heparin and LMWH inhibit the activation of Factor IIa and Factor Xa, respectively.^25^ For this reason, heparin‐based therapies may be not suitable for the CVT patients with AT‐III deficiency. Furthermore, although heparinoids have the capability of preventing further thrombus extension, existing thrombus is not readily lysed.^26^, ^27^, ^28^ Given the disadvantages of anticoagulation, it is proposed that thrombolysis in CVT patients may produce desirable clinical benefits.^6^, ^8^ Thrombolytic agents directly influence the fibrinolytic system and convert plasminogen to plasmin, which eventually removes the blood clot in the circulation. Although local thrombolysis has shown benefits in some CVT cases, this invasive therapy inevitably faces a risk of hemorrhagic transformation along with poor clinical outcomes.^9^ Invasive strategies such as local thrombolysis and mechanical thrombectomy are limited to observational studies.^6^, ^7^, ^8^, ^9^, ^10^ Therefore, endovascular mechanical thrombectomy or thrombolysis will be considered only at the condition of CVT‐associated clinical deterioration.

**Figure 3:**
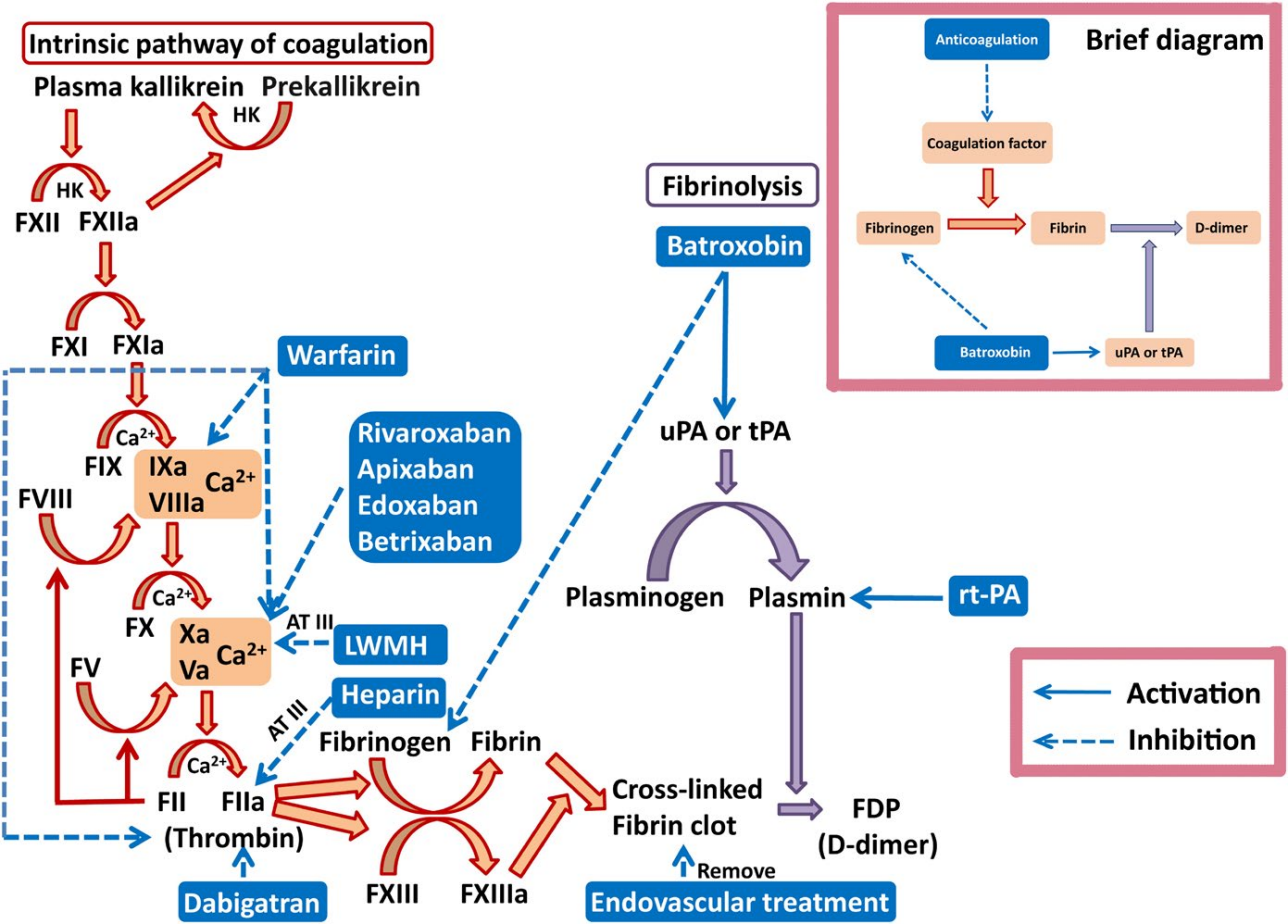
The pattern diagram of coagulation‐fibrinolysis pathways: Anticoagulation such as LWMH mainly inhibits coagulation pathway; tPA dissolves the thrombus in venous sinus, which acts on fibrinolysis pathway; endovascular treatment removes the thrombus directly; Batroxobin shares a combinatory effect of anticoagulation and fibrinolysis. The brief diagram is shown in the upper right with a purple frame

Batroxobin, an agent widely used in arterial thrombosis, binds fibrinogen with 10‐fold affinity as compared with thrombin, followed by a decrease in the level of fibrinogen in the circulation, resulting in the inhibition of fibrin formation and thrombus elongation.^29^ Moreover, it has been demonstrated that batroxobin could prompt the production of tissue plasminogen activator (tPA), mitigate the activation of tissue plasminogen inhibitor (PAI), impede platelet activation, and reduce blood viscosity improving microcirculation.^30^, ^31^, ^32^ The coagulation‐fibrinolysis pathways and the target of the aforementioned interventions are presented in Figure 3.

In the previous publication, it has been verified that batroxobin may be a promising adjunct for standard anticoagulation strategy of CVT control; however, the MRV/CTV utilized was a rough imaging technique for evaluating the follow‐up imaging outcomes.^17^ In order to further enhance the accuracy of the assessment and provide a more convincing conclusion, we utilized MRBTI as the main imaging evaluation tool in this study. Yang et al found MRBTI can confirm the diagnosis of CVT with a high accuracy by which thrombus in the veins/sinuses can be directly visualized.^18^ Instead of directly imaging thrombus of MRBTI, MRV relies on the presentation of altered blood flow in veins, which may not express the real luminal patency and compromise the evaluating confidence to some extent.

In agreement with the previous results, this cohort of batroxobin‐treated patients experienced improved recanalization rates and degree on TOF MRV and MRBTI. The reduction in stenosis extent on MRBTI in batroxobin group was more significant than that in control. Among the patients with normal fibrinogen, batroxobin in combination with anticoagulation still exhibited more benefit than anticoagulation alone. Although we cannot perform comparisons among patients with high fibrinogen levels, the recent study showed the important role of batroxobin in CVT with high fibrinogen levels.^16^ The stenosis improvement rates on MRBTI maps were more remarkable in the batroxobin group compared to the control group. These results indicate some degree of lysis is taking place or enhanced vessel remodeling in the batroxobin group. Although the stenosis extent at follow‐up between the two groups did not reach a significant difference, changes in the stenosis extent after intravenous batroxobin showed strong trends on dissolving thrombus. The MRBTI map gave us direct visualization of clot elimination with nonionizing radiation suggesting this may be a new noninvasive accurate way of following CVT patients. The elevation of D‐dimer (more than 20‐fold of baseline) and reduction in fibrinogen after batroxobin use presented in the previous study provide a convincing demonstration that fibrinolysis and thrombolysis induced by batroxobin can dissolve CVT thrombus.^16^

Subgroup analysis based on the nine subtypes of venous system segments was also performed. Only the superior sagittal segment displayed remarkable stenosis reduction in batroxobin group compared with control. Small sample sizes constricted the test power, despite which, we still thought that the thrombus in superior sagittal segment might be more susceptible to defibrase drugs. This may be explained by more drug reaching this area first given normal drainage patterns.

NIHSS scores and mRS scores at discharge and 6 months were equivalent between the patients with batroxobin use and controls in the previous study. Only discharge NIHSS scores were significantly better in batroxobin group.^16^ The current study revealed no significant differences in NIHSS or mRS at follow‐up. As opposed to cerebral arterial occlusion, the neurological deficits mediated by CVT can be more asymptomatic, the NIHSS scores and mRS scores may not accurately reflect the differences in clinical outcomes of patients with CVT (especially in regard to headaches, papilledema, venous hypertension all related to chronic venous drainage insufficiency). One patient suffered from cerebral hemorrhage at admission, after anticoagulation treatment, the hematoma did not further enlarge. No other hemorrhages were noted in this study. We cannot conclude on the safety of batroxobin in this study, but in the previously published work, the risk of hemorrhage in batroxobin group was lower than control but with nonstatistical significance.^16^ Other previous reports regarding batroxobin treating cerebral arterial disease have found a low risk of batroxobin‐induced hemorrhage.^33^, ^34^ Therefore, we believe that using batroxobin in CVT patients is safe.

There were some limitations to this study. Our small sample size renders the study underpowered and restricts the conclusion reliability, due to which, the multivariate analysis could not involve enough relative confounding factors and subgroup analysis may not suffice achieving convincing results. Large randomized trials would be difficult with such a rare disease process. Despite this, a well‐designed large sample size trial is needed to further evaluate. The present study is a real‐world experience, and this means it cannot be randomized and controlled. The low‐quality design may affect the study conclusion. The treatment group was assigned in accordance with the patients consent and wishes to be treated with this novel drug and controls identified. Cohort studies such as ours can be valuable despite not being randomized. Because most of the enrolled patients only conducted two times of MRBTI in this study, more sequential MRBTI imaging would be necessary to evaluate if batroxobin was able to accelerate the recanalization in CVT patients. Although the follow‐up time between the two groups did not reach difference and multivariate analysis had adjusted for this confounding factor, the bias still existed in this study and the conclusions need to be further determined.

## 5 CONCLUSION

With the help of MRBTI, we notice that batroxobin may promote recanalization and attenuate venous sinus stenosis in CVT. Considering the limitations of this study, multicenter, well‐designed, large sample size prospective trials are needed to further verify this conclusion.

## CONFLICT OF INTEREST

All authors report no conflicts of interest.
